# The relationship between the dietary index based Meiji nutritional profiling system for adults and lifestyle-related diseases: a predictive validity study from the National Institute for Longevity Sciences—Longitudinal Study of Aging

**DOI:** 10.3389/fnut.2024.1413980

**Published:** 2024-07-03

**Authors:** Tao Yu, Shu Zhang, Ryota Wakayama, Tomohito Horimoto, Chikako Tange, Yukiko Nishita, Rei Otsuka

**Affiliations:** ^1^R&D Division Meiji Co., Ltd., Tokyo, Japan; ^2^National Center for Geriatrics and Gerontology, Aichi, Japan

**Keywords:** nutrient profiles, nutritional profiling model, nutritional quality, product reformulation, healthy food, healthy society, non-communicable diseases

## Abstract

**Background:**

Nutritional profiling system (NPS) holds promise as a public health tool for companies to measure product healthiness and for individuals in making healthier food choices. The Meiji NPS for adults specifically targets lifestyle-related diseases prevalent among Japan’s adult population, including overweight/obesity, hypertension, diabetes, and dyslipidemia. This study examined the cross-sectional association between the Meiji NPS for adults Dietary Index (MNfA-DI) and indicators of lifestyle-related diseases in a population.

**Methods:**

The study comprised 1,272 middle-aged individuals (40–64 years, 50.1% male) who participated in the seventh wave (2010–2012) of the National Institute for Longevity Sciences—Longitudinal Study of Aging project, with no missing data on three-day dietary records. The MNfA-DI was computed at the individual diet level (accounting for the whole diet) using arithmetic energy-weighted means. A higher MNfA-DI indicated a greater nutritional quality of an individual’s overall diet. Lifestyle-related disease indicators included body mass index (BMI, kg/m^2^), body fat (%), systolic and diastolic blood pressure (mmHg), fasting plasma glucose (mg/dL), HbA1c (%), triglyceride levels (mg/dL), LDL, and HDL cholesterol levels (mg/dL). A multiple regression model was used to assess the association between the MNfA-DI and lifestyle-related disease indicators, adjusting for demographics, socioeconomic status, lifestyle factors, disease history, and energy intake as covariates, depending on the outcome.

**Results:**

The median (interquartile range) age and MNfA-DI were 53.0 (46.0, 59.0) years and 10.1 (6.0, 14.0) points, respectively. MNfA-DI was negatively associated with body fat [partial regression coefficient (95% confidence interval) −0.04 (−0.07, −0.01)], diastolic blood pressure [−0.08 (−0.17, −0.002)], fasting plasma glucose [−0.18 (−0.33, −0.01)], and triglyceride [−1.36 (−2.16, −0.55)]. Additionally, MNfA-DI was also associated with almost indicators (except for LDL and HDL cholesterol) among participants with a BMI between 18.5 and 24.9 kg/m^2^.

**Conclusion:**

These findings suggest that the Meiji NPS for adults could be associated with a lower risk of lifestyle-related diseases. In addition, from a public health nutrition perspective, the Meiji NPS for adults may be useful to assess the food healthiness of the adult population.

## 1 Introduction

Japan is often recognized worldwide for healthy eating habits, characterized by a relatively balanced diet and low-fat intake (1–3). However, according to the Global Burden of Disease Report (GBD) 2017, approximately 30% of mortality is attributable to diet (excessive salt consumption and inadequate intake of fruits, whole grains, nuts, and seaweed) in East Asia, including Japan, which is higher than the global average of 22% (4). Rigorous research from Japan recommends increasing calcium, dietary fiber, iron, and potassium intake (5). Despite Japan’s reputation as one of the healthiest dietary nations in the world, there are many opportunities for improvement (6, 7). According to the GBD Study 2019, unhealthy eating habits rank as the third most important risk factor for death in Japan and remain a factor that needs to be improved (8). Lifestyle-related diseases are defined as a group of diseases (such as overweight/obesity, hypertension, diabetes and hyperlipidemia) in which lifestyle habits such as dietary habits, exercise habits, rest, smoking, and alcohol consumption are involved in the development and progression of the disease, and the concept is similar to non-communicable diseases (in Japan, they are sometimes referred to as lifestyle-related diseases, a group of diseases excluding chronic obstructive pulmonary disease from non-communicable diseases) (9–11). These diseases are acknowledged as critical health concerns, especially in adulthood, echoing global trends (6, 12–17). Data from a survey performed on 2,467 people across 300 randomly selected districts in Japan (National Health and Nutrition Examination Survey, called NIPPON data80) revealed that approximately 40% of Japanese adults experienced hypertension, more than 10% presented diabetes, 41% had hypercholesterolemia, and approximately 7% presented low HDL cholesterol (18, 19).

In this context, European countries have implemented front-of-pack labeling (FoPL) to promote healthier diets, and the core concept is the Nutrition Profiling Systems (NPS) (20–24). According to the World Health Organization definition, nutritional profiling is defined as “the science of categorizing or ranking foods according to their nutritional composition.” In other words, the NPS allow various foods and products to be characterized as healthy or unhealthy according to predefined criteria, and the FoPL aims to contribute to the food environment and people’s eating behavior (25–28). In some studies, FoPL has potentially guided people’s purchasing behavior toward healthier products, with its potential usefulness being discussed (29–32).

Food companies are encouraged to develop NPS for product reform, which can directly contribute to people’s health (33–37). Whether the NPS used for FoPL or the NPS developed by companies, the aims are different, but the contribution to people’s health challenges is no different. Besides creating an NPS, NPS creator are required to verify whether the developed NPS are related to the health outcomes they previously set, which could be considered of even greater importance (23, 38, 39). The validity of some NPS, such as the Nutri-score and healthy star rating (HSR), is supported by several studies using established NPS, which have been carefully examined in relation to non-communicable diseases and clinical data representative of each disease (38–40). However, the use of established overseas NPS could not consider the differences in eating habits and health issues between overseas and Japan, and the possibility of misclassification of healthiness in foods cannot be ignored (41–44). Despite lifestyle-related diseases being an important health issue in Japan, there are no NPS that have been confirmed to be associated with health outcomes in Japan worldwide. Therefore, Meiji developed the Meiji NPS for adults, focusing on the health issues (lifestyle-related diseases) of the Japanese adult population.

This study aimed to confirm the predictive validity of the Meiji NPS for adults by cross-sectionally examining its association with clinical indicators for each lifestyle-related disease from an epidemiological perspective. Confirming the predictive validity of the Meiji NPS for adults will enhance its credibility and ensure its validity in the targeted age group.

## 2 Materials and methods

### 2.1 Study population

This study is a cross-sectional study comprised data from the seventh wave of the National Institute for Longevity Sciences-Longitudinal Study of Aging (NILS-LSA), which was conducted between July 2010 and July 2012 (45, 46). The NILS-LSA is an ongoing longitudinal cohort study that involves a wide range of fields related to aging and geriatric diseases, including medicine, psychology, exercise, body composition, and nutrition. Various experts collaborated to conduct detailed surveys (basic questionnaires, medical examinations, and anthropometric, physical, and nutritional assessments), data collection, and analyses. Participants of the NILS-LSA were randomly selected based on age and sex, from the local populations of Obu City and Higashiura Town, in the neighborhood of the National Center for Geriatrics and Gerontology in Aichi Prefecture, Japan. Among the 1,369 participants (aged 40–64 years) in the seventh wave (2010–2012) survey, 97 (7.1%) had missing data on 3-day dietary records. For power and study design, those with missing outcome variables (lifestyle-related disease indicators) were not excluded. The reason was that the study was designed as an exploratory study examining associations with multiple outcomes, and therefore it was not considered necessary to limit the participants with all outcomes (47).

### 2.2 Nutritional assessments

Participants completed a three-day dietary record in order to assess food consumption and nutrient intakes (48). Food records were completed over three consecutive days (two weekdays and one weekend day) since eating habits differ between weekdays and weekends. Food items were weighed individually using a scale (1-kg kitchen scales, Sekisui Jushi, Tokyo, Japan) prior to cooking or with portion sizes estimated. Additionally, participants documented their dietary intake using a disposable camera (27 shots, Fuji Film, Tokyo, Japan), capturing images of meals before and after consumption. Dietitians utilized these photographs to address any missing data and contacted participants via telephone to resolve discrepancies or gather additional information as needed. Mean nutrient and energy intake over a 3-day period were calculated based on the Standard Tables of Food Composition 2010 in Japan and other relevant sources (48). After categorizing food items following the Japan National Health and Nutrition Examination Survey (e.g., legumes, seeds, vegetables, fruits, and dairy), the food items included in each food group were carefully rechecked to match the assessment items in the Meiji NPS (49). Food items with significantly different nutritional characteristics were subsequently excluded from the corresponding food group. For instance, cream or milk coffee were excluded from dairy and dairy products group. This approach supports the validity of the Meiji NPS scoring for adults. Finally, the total consumption of each food group was determined by aggregating the consumption of all foods within the group. For dried foods (e.g., dried *wakame*), their actual weight after water absorption was used. However, the total nutrient intakes for dried foods were calculated based on their dried state.

### 2.3 Meiji NPS for adults and Meiji NPS for adults dietary index (MNfA-DI)

The Meiji NPS for adults considers differences in eating habits between Japan and other countries, as well as differences in the applicability of epidemiological studies (50). It places significant emphasis on evidence from the Japanese population, particularly in addressing lifestyle-related diseases as a major health concern for adults. The Meiji NPS for adults includes recommended nutritional intake levels such as protein, dietary fiber, calcium, iron, and vitamin D, along with recommended food groups, including fruits, vegetables, nuts, legumes, and dairy. Additionally, it outlines restricted nutrient intake, including energy, saturated fatty acids, sugar (glucose, fructose, galactose, sucrose, maltose, and lactose), and salt equivalents. Detailed protocols for the creation of the Meiji NPS for adults and its correlation with existing NPS are available in other studies (50). The study initially assessed each food consumed by the participants using the Meiji NPS for adults. The Meiji NPS for Adults Dietary Index (MNfA-DI) was then calculated using the following formula (20).


$$\mathrm{Dietary}\;\mathrm{index}=\frac{\sum_{i=1}^{n}\mathrm{FSiEi}}{\sum_{i=1}^{n}Ei}$$

where i is the food (or beverage) consumed by the participant, FSi is the food (or beverage) score. Score is determined by adding or subtracting points for items listed in the Meiji NPS for adults. Ei is the mean daily energy intake from the food (or beverage), and n is the number of different foods consumed. A higher MNfA-DI reflects the higher nutritional quality of an individual’s overall diet.

### 2.4 Lifestyle-related diseases indicators

Lifestyle-related diseases in this study were limited to four diseases closely related to diet: overweight/obesity, hypertension, type 2 diabetes, and dyslipidemia. BMI (kg/m^2^) and body fat (%) were used as indicators of overweight/obesity, systolic and diastolic blood pressure (mmHg) as indicators of hypertension, fasting blood glucose (mg/dL) and HbA1c (%) as indicators of type 2 diabetes, triglycerides (mg/dL), LDL and HDL cholesterol (mg/dL), which were employed as outcomes of the study, respectively (LDL cholesterol is calculated using the Friedewald formula. That is, LDL cholesterol = total cholesterol − HDL cholesterol − triglycerides/5) (51). Weight and height were measured in the fasting state (approximately 9–10 a.m.) to the nearest 0.1 kg and 0.1 cm, respectively, with participants wearing light clothing and no shoes.

### 2.5 Other variables

Self-administered questionnaires were used to assess marital status (never married/ married/ divorced, bereaved, separate), household income (10 levels), years of education, living situation (living alone or no), occupation (not employed, housewife or house husband, employed, other), smoking status (never, stopped, smoking), depression status (assessment by the Center for Epidemiologic Studies Depression Scale). Participants’ daily steps were recorded using a uniaxial accelerometer (Lifecorder; Suzuken, Aichi, Japan). Participants wore the accelerometer for seven consecutive days (sleeping, bathing, or traveling were not included in the seven-day measurement duration). The average number of steps taken over the 7-day period was employed for this study.

The BMI was used as the criterion for determining overweight, even if the BMI ≥ 25 kg/m^2^. The blood pressure (systolic and diastolic) obtained from that measurement was used to identify the presence of the abnormal of blood pressure (defined as a systolic blood pressure ≥ 140 mmHg, or diastolic blood pressure ≥ 85 mmHg). Abnormal blood glucose was determined using fasting plasma glucose and HbA1c, with fasting plasma glucose ≥126 mg/dL or HbA1c ≥ 6.0%. Lipids were considered abnormal if the TG ≥ 140 mg/dL or the HDL < 40 mg/dL. Lifestyle-related disease scores were created from overweight, abnormal blood pressure, abnormal blood glucose and abnormal lipids (0–4) to characterize the population (mainly used in Table 1). The lifestyle-related disease score was also created with continuous variables of abnormal blood pressure, above blood glucose and abnormal lipids (0–3) as adjustment variables (mainly used in Tables 2, 3).

**Table 1:** The characteristics of all participants and in the three groups of MNfA-DI.

	All participants 10.1 (6.0 – 14.0) (*n* = 1272)	Low group 4.5 (2.5 – 6.0) (*n* = 424)	Medium group 10.1 (8.8 – 11.3) (*n* = 424)	High group 15.9 (14.0 – 18.8) (*n* = 424)	*p*-value
Sex					
Male	637 (50.1%)	204 (48.1%)	184 (43.4%)	249 (58.7%)	<0.001
Female	635 (49.9%)	220 (51.9%)	240 (56.6%)	175 (41.3%)	
Age	53.0 (46.0–59.0)	51.0 (46.0–56.5)	53.0 (46.0–59.0)	55.5 (47.0–61.0)	<0.001
CES-D	5.0 (2.0–10.0)	5.0 (2.0–10.5)	5.0 (1.0–10.0)	4.0 (1.0–9.5)	0.07
Marital status					
Unmarried	67 (5.3%)	26 (6.1%)	9 (2.1%)	32 (7.5%)	<0.01
Married	1,130 (88.8%)	372 (87.7%)	389 (91.7%)	369 (87.0%)	
Divorce, bereavement, or separate	75 (5.9%)	26 (6.1%)	26 (6.1%)	23 (5.4%)	
Household income (*n* = 1,264)					
Low	521 (41.2%)	170 (40.4%)	175 (41.6%)	176 (41.7%)	0.66
Medium	454 (35.9%)	145 (34.4%)	158 (37.5%)	151 (35.8%)	
High	289 (22.9%)	106 (25.2%)	88 (20.9%)	95 (22.5%)	
Education years	14.0 (12.0–16.0)	14.0 (12.0–16.0)	14.0 (12.0–16.0)	13.0 (12.0–16.0)	0.66
Living alone	59 (4.6%)	21 (5.0%)	14 (3.3%)	24 (5.7%)	0.25
Occupation					
Unemployed, housewife or husband	222 (17.5%)	57 (13.4%)	85 (20.0%)	80 (18.9%)	0.02
Employed	918 (72.2%)	332 (78.3%)	291 (68.6%)	295 (69.6%)	
Other	132 (10.4%)	35 (8.3%)	48 (11.3%)	49 (11.6%)	
Daily steps (*n* = 1,235)	8,981 (6,950–11,264)	8,960 (6,882–11,213)	8,643 (6,753–10,954)	9,265 (7,232–11,476)	0.03
Smoking status					
Never	747 (58.7%)	213 (50.2%)	274 (64.6%)	260 (61.3%)	<0.001
Stopped	337 (26.5%)	113 (26.7%)	102 (24.1%)	122 (28.8%)	
Smoking	188 (14.8%)	98 (23.1%)	48 (11.3%)	42 (9.9%)	
Alcohol volume	1.5 (0.4–15.1)	4.9 (0.4–24.9)	1.2 (0.4–10.4)	1.4 (0.4–10.8)	<0.001
MNfA-DI	10.1 (6.0–14.0)	4.5 (2.5–6.0)	10.1 (8.8–11.3)	15.9 (14.0–18.8)	<0.001
lifestyle-related disease score (*n* = 1,246)					
0	850 (68.2%)	286 (68.9%)	283 (67.7%)	281 (68.0%)	0.74
1	344 (27.6%)	115 (27.7%)	114 (27.3%)	115 (27.8%)	
2	50 (4.0%)	14 (3.4%)	20 (4.8%)	16 (3.9%)	
3	1 (0.1%)	0 (0.0%)	1 (0.2%)	0 (0.0%)	
4	1 (0.1%)	0 (0.0%)	0 (0.0%)	1 (0.2%)	

Quantitative variables are presented as median (interquartile range) and categorical variables as number of people (percentage). A valid number is indicated below each item only for items with missing data. The chi-square test (for categorical variables) or Kruskal–Wallis test (for quantitative variables) were used to test for differences in each characteristic according to high, medium, and low MNfA-DI. MNfA-DI, Meiji NPS for adults Dietary Index.

**Table 2:** Association between the MNfA-DI (continuous variable) and each lifestyle-related disease indicators.

	Model 1			Model 2		
	B	95% CI	*p*-value	B	95% CI	*p*-value
BMI (kg/m^2^, *n* = 1,203)	−0.02	−0.05, 0.01	0.11	−0.02	−0.05, 0.01	0.11
Body fat (%, *n* = 1,198)	−0.07	−0.11, −0.03	<0.001	−0.04	−0.07, −0.01	<0.01
Systolic blood pressure (mmHg, *n* = 1,224)	−0.14	−0.28, −0.002	<0.05	−0.10	−0.23, 0.03	0.12
Diastolic blood pressure (mmHg, *n* = 1,224)	−0.11	−0.20, −0.03	0.01	−0.08	−0.17, −0.002	<0.05
Fasting plasma glucose (mg/dL, *n* = 1,206)	−0.23	−0.37, −0.08	<0.01	−0.18	−0.33, −0.01	0.02
HbA1c (%, *n* = 1,227)	−0.003	−0.01, 0.001	0.15	−0.002	−0.01, 0.002	0.33
Triglycerides (mg/dL, *n* = 1,206)	−1.62	−2.42, −0.82	<0.001	−1.36	−2.16, −0.55	<0.001
LDL-cholesterol (mg/dL, *n* = 1,206)	0.27	−0.01, 0.54	0.06	0.23	−0.05, 0.50	0.11
HDL-cholesterol (mg/dL, *n* = 1,227)	−0.02	−0.15, 0.12	0.81	−0.09	−0.22, 0.04	0.18

For BMI, Model 1 was adjusted for age, sex, and energy intake. Model 2 included household income, education year, marital status, occupation, smoking status, daily steps, and lifestyle-related disease score into Model 1. For body fat, Model 1 was adjusted for age, sex, and energy intake. Model 2 included BMI, household income, education years, marital status, occupation, smoking status, daily step, and lifestyle-related disease score to Model 1. For each lifestyle-related disease indicator, Model 1 was adjusted for age, sex, and energy intake. Model 2 included household income, education years, marital status, occupation, smoking status, and daily steps in addition to Model 1. B, Partial regression coefficient; CI, confidence interval; MNfA-DI, Meiji NPS for adults Dietary Index.

**Table 3:** Association between the MNfA-DI (continuous variable) and each lifestyle-related disease indicators in participants with BMI between 18.5 and 24.9 kg/m^2^.

	B	95% CI	*p*-value
BMI (kg/m^2^, *n* = 897)	−0.03	−0.05, −0.02	<0.001
Body fat (%, *n* = 894)	−0.06	−0.11, −0.02	0.003
Systolic blood pressure (mmHg, *n* = 912)	−0.20	−0.37, −0.04	0.02
Diastolic blood pressure (mmHg, *n* = 912)	−0.14	−0.24, −0.03	0.009
Fasting plasma glucose (mg/dL, *n* = 900)	−0.31	−0.49, −0.14	<0.001
HbA1c (%, *n* = 900)	−0.002	−0.004, −0.001	0.04
Triglycerides (mg/dL, *n* = 900)	−1.73	−2.67, −0.79	<0.001
LDL-cholesterol (mg/dL, *n* = 900)	0.04	−0.29, 0.37	0.82
HDL-cholesterol (mg/dL, *n* = 915)	−0.04	−0.21, 0.13	0.66

For BMI, Model was adjusted for age, sex, energy intake, household income, education year, marital status, occupation, smoking status, daily steps, and lifestyle-related disease score. For body fat, Model was adjusted for age, sex, energy intake, household income, education year, marital status, occupation, smoking status, daily steps, and lifestyle-related disease score. For each lifestyle-related disease indicator, Model was adjusted for age, sex, energy intake, household income, education year, marital status, occupation, smoking status, daily steps. B, Partial regression coefficient; CI, confidence interval; MNfA-DI Meiji NPS for adults Dietary Index.

### 2.6 Statistical analysis

The Kruskal–Wallis test was used to examine differences in each characteristic and nutrient intake status across high, medium, and low MNfA-DI categories. A multiple regression model was used to assess the association between MNfA-DI and lifestyle-related disease indicators while considering demographics, socioeconomic status, lifestyle factors, disease history, and energy intake as covariates, contingent upon the outcome. For BMI, Model 1 was adjusted for age, sex, and energy intake, while Model 2 included household income (the tier from 1 to 10 is divided into low, medium, and high group), education years, marital status, occupation, smoking status, daily steps, and lifestyle diseases score in addition to Model 1. Regarding body fat, Model 1 was adjusted for age, sex, and energy intake, while Model 2 included BMI, household income, education years, marital status, occupation, smoking status, daily steps, and lifestyle diseases score besides Model 1. For each lifestyle-related disease indicator, Model 1 was adjusted for age, sex, and energy intake, and Model 2 included household income, education years, marital status, occupation, smoking status, and daily steps in addition to Model 1. The hypothesis in this analysis is that the MNfA-DI will be significantly negatively associated with each of the lifestyle disease indicators.

All statistical analyses were performed using STATA version 16.0 (Stata Corp LLC, Texas, United States). All reported *p*-values were two-sided, and a *p*-value <0.05 was considered significant.

### 2.7 Ethics statement

The NILS-LSA was approved by the Conflict of Interest and Ethics Review at the National Center for Geriatrics and Gerontology, and written informed consent was obtained from all participants (No. 1665–2). The conduct of this study was approved by the Conflict of Interest and Ethics Review at the National Center for Geriatrics and Gerontology (No. 1757).

## 3 Results

This study included 1,272 individuals, with a median age of 53.0 (46.0–59.0) years and an MNfA-DI of 10.1 (6.0–14.0) points; 88.8% of the individuals were married, and 82.6% were working. Household income was divided into three groups, with 41.2% belonging to the low group and 22.9% to the high group. The median number of daily steps was 8,981, and 14.8% of individuals reported being smokers. Additionally, 68.2% of participants had no lifestyle-related disease score. Significant differences were observed in sex, age, marital status, occupation, daily steps, smoking status, and alcohol volume across high, medium, and low MNfA-DI scores. Detailed information on the analyzed population is shown in Table 1.

Table 4 shows the differences in unadjusted nutrient and food intake across low, medium, and high MNfA-DI, while Table 5 shows the differences for each nutrient and food adjusted by the density method according to low, medium, and high MNfA-DI. Most nutrients and foods showed significant differences among the low, medium, and high MNfA-DI. The salt intake showed significantly higher values across MNfA-DI categories in Table 4, while Table 5 shows fewer differences. In addition, the intake of nuts showed no significant differences either with or without energy adjustment.

**Table 4:** Differences in nutrient and food intake (raw data) in low, medium, and high MNfA-DI groups.

		Low group 4.5 (2.5 – 6.0) (*n* = 424)	Medium group 10.1 (8.8 – 11.3) (*n* = 424)	High group 15.9 (14.0 – 18.8) (*n* = 424)	*p*-value
EN	Kcal	1907 (1680–2,215)	1838 (1618–2,134)	2019 (1795–2,295)	<0.001
Protein	g	68.8 (58.6–79.4)	72.1 (64.0–80.8)	80.0 (69.7–92.1)	<0.001
Fat	g	63.3 (52.6–75.5)	59.8 (50.1–71.3)	61.7 (50.8–72.0)	0.01
SFA	g	18.9 (14.2–23.4)	17.4 (14.0–21.5)	17.4 (14.4–21.5)	0.007
n6 PUFA	g	10.3 (8.6–12.6)	9.9 (8.1–12.2)	10.1 (8.1–12.5)	0.14
n3 PUFA	g	2.2 (1.6–2.8)	2.3 (1.7–2.9)	2.5 (1.8–3.2)	<0.001
Carbohydrate	g	257.0 (225.4–301.0)	261.6 (231.8–301.0)	295.7 (258.1–338.7)	<0.001
Sugar	g	46.8 (34.2–60.6)	46.6 (35.3–60.1)	50.7 (35.9–65.0)	0.06
Fiber	g	17.2 (14.6–19.9)	18.8 (15.9–22.0)	21.2 (18.4–24.6)	<0.001
VA	μgRAE	407 (306–572)	466 (343–651)	552 (407–745)	<0.001
VD	μg	4.7 (3.1–7.3)	6.0 (3.7–9.3)	7.3 (4.7–10.9)	<0.001
VE	mg	7.2 (6.0–8.7)	7.1 (5.8–8.8)	7.6 (6.3–9.2)	0.006
VK	μg	173 (125–233)	198 (135–268)	226 (162–318)	<0.001
VB1	mg	0.9 (0.7–1.0)	0.9 (0.8–1.1)	1.0 (0.8–1.2)	<0.001
VB2	mg	1.1 (0.9–1.4)	1.2 (1.0–1.4)	1.4 (1.2–1.6)	<0.001
Niacin	mgNE	15 (12–18)	17 (14–20)	18 (15–22)	<0.001
VB6	mg	1.1 (0.9–1.3)	1.2 (0.9–1.4)	1.4 (1.1–1.6)	<0.001
VB12	μg	4.9 (3.1–7.8)	5.4 (3.7–8.3)	6.5 (4.2–10.0)	<0.001
Folic acid	μg	276 (231–353)	309 (249–370)	339 (276–423)	<0.001
Pantothenic acid	mg	5 (4–6)	5 (5–6)	6 (5–7)	<0.001
VC	mg	87 (60–123)	98 (71–136)	112 (77–155)	<0.001
Potassium	mg	2,236 (1898–2,595)	2,428 (2067–2,831)	2,738 (2358–3,202)	<0.001
Calcium	mg	454 (360–565)	524 (412–625)	630 (495–747)	<0.001
Magnesium	mg	239 (204–282)	256 (220–301)	297 (251–346)	<0.001
Phosphorous	mg	964 (802–1,100)	1,013 (899–1,147)	1,162 (1008–1,332)	<0.001
Iron	mg	7.3 (6.2–8.6)	7.8 (6.6–9.1)	8.8 (7.5–10.4)	<0.001
Zinc	mg	7.4 (6.2–8.8)	8.0 (7.0–9.3)	9.0 (7.8–10.5)	<0.001
Copper	mg	1.0 (0.9–1.2)	1.1 (0.9–1.3)	1.3 (1.1–1.5)	<0.001
Sodium	g	10.1 (8.4–11.7)	10.0 (8.3–11.7)	10.5 (9.0–12.1)	<0.001
Fruits	g	66.7 (18.2–125.3)	88.5 (37.8–163.9)	113.7 (36.7–202.0)	<0.001
Vegetables	g	255.7 (185.8–336.5)	287.8 (209.5–368.7)	317.7 (235.9–423.5)	<0.001
Nuts	g	0.3 (0.0–2.9)	0.7 (0.0–3.1)	0.9 (0.0–3.3)	0.08
Legumes	g	30.7 (13.4–59.6)	49.0 (20.4–79.7)	64.2 (33.2–110.5)	<0.001
Dairy	g	21.8 (0.0–69.2)	66.7 (6.7–155.3)	133.3 (15.8–201.8)	<0.001

The Kruskal–Wallis test was used to test the differences in each characteristic and nutrient intake status according to high, medium, and low MnfA-DI. All variables are presented as median (inter-quartile range). Sugar indicates the total value of glucose, fructose, galactose, sucrose, maltose and lactose. MnfA-DI, Meiji NPS for adults Dietary Index.

**Table 5:** Differences in nutrient and food intake (density adjusted) in low, medium, and high MNfA-DI groups.

		Low group 4.5 (2.5 – 6.0) (*n* = 424)	Medium group 10.1 (8.8 – 11.3) (*n* = 424)	High group 15.9 (14.0 – 18.8) (*n* = 424)	*p*-value
EN	Kcal	1,907 (1680–2,215)	1,838 (1,618–2,134)	2,019 (1,795–2,295)	<0.001
Protein	% EN	14.3 (13.2–15.7)	15.4 (14.2–16.7)	15.8 (14.5–17.3)	<0.001
Fat	% EN	30.3 (26.6–33.7)	28.9 (25.6–32.4)	27.4 (24.0–30.4)	<0.001
SFA	% EN	8.8 (7.2–10.5)	8.3 (7.0–10.0)	7.8 (6.5–9.3)	<0.001
n6 PUFA	% EN	4.8 (4.2–5.7)	4.8 (4.2–5.5)	4.5 (3.9–5.3)	<0.001
n3 PUFA	% EN	1.0 (0.8–1.3)	1.1 (0.9–1.3)	1.1 (0.8–1.4)	0.002
Carbohydrate	% EN	54.4 (49.6–58.9)	57.1 (53.7–61.2)	59.0 (54.5–62.6)	<0.001
Sugar	% EN	10.0 (7.3–12.5)	10.2 (7.6–12.8)	9.9 (7.4–13.0)	0.55
Fiber	g/1,000 Kcal	9.0 (7.8–10.3)	10.0 (8.8–11.2)	10.5 (9.1–11.9)	<0.001
VA	μgRAE/1,000 Kcal	217 (159–294)	253 (185–334)	275 (203–367)	<0.001
VD	μg/1,000 Kcal	2.4 (1.6–3.7)	3.2 (2.0–4.8)	3.6 (2.3–5.4)	<0.001
VE	mg/1,000 Kcal	3.8 (3.2–4.4)	3.9 (3.2–4.5)	3.8 (3.1–4.5)	0.38
VK	μg/1,000 Kcal	88 (66–120)	103 (72–138)	111 (81–156)	<0.001
VB1	mg/1,000 Kcal	0.4 (0.4–0.5)	0.5 (0.4–0.6)	0.5 (0.4–0.6)	<0.001
VB2	mg/1,000 Kcal	0.6 (0.5–0.7)	0.7 (0.6–0.8)	0.7 (0.6–0.8)	<0.001
Niacin	mgNE/1,000 Kcal	8 (7–9)	9 (7–10)	9 (8–11)	<0.001
VB6	mg/1,000 Kcal	0.6 (0.5–0.7)	0.6 (0.5–0.7)	0.7 (0.5–0.8)	<0.001
VB12	μg/1,000 Kcal	2.5 (1.7–4.0)	2.9 (2.0–4.4)	3.1 (2.1–5.0)	<0.001
Folic acid	μg/1,000 Kcal	145 (123–172)	160 (134–195)	169 (140–210)	<0.001
Pantothenic acid	mg/1,000 Kcal	3 (2–3)	3 (3–3)	3 (3–3)	<0.001
VC	mg/1,000 Kcal	46 (31–64)	52 (37–73)	55 (40–79)	<0.001
Potassium	mg/1,000 Kcal	1,147 (1010–1,333)	1,295 (1124–1,481)	1,362 (1171–1,602)	<0.001
Calcium	mg/1,000 Kcal	234 (187–292)	280 (221–339)	312 (240–384)	<0.001
Magnesium	mg/1,000 Kcal	123 (111–141)	136 (120–153)	145 (129–169)	<0.001
Phosphorous	mg/1,000 Kcal	495 (445–540)	545 (493–600)	579 (514–646)	<0.001
Iron	mg/1,000 Kcal	3.8 (3.3–4.4)	4.1 (3.6–4.8)	4.3 (3.7–5.1)	<0.001
Zinc	mg/1,000 Kcal	3.9 (3.4–4.3)	4.3 (3.9–4.7)	4.5 (4.1–4.9)	<0.001
Copper	mg/1,000 Kcal	0.5 (0.5–0.6)	0.6 (0.5–0.7)	0.6 (0.6–0.7)	<0.001
Sodium	g/1,000 Kcal	5.2 (4.5–5.8)	5.3 (4.7–6.0)	5.3 (4.6–5.9)	0.22
Fruits	g/1,000 Kcal	34.3 (9.0–64.7)	49.6 (20.7–88.0)	54.4 (19.2–103.3)	<0.001
Vegetables	g/1,000 Kcal	130.7 (97.8–169.9)	154.9 (112.2–196.0)	156.1 (117.0–217.1)	<0.001
Nuts	g/1,000 Kcal	0.2 (0.0–1.6)	0.3 (0.0–1.6)	0.4 (0.0–1.6)	0.14
Legumes	g/1,000 Kcal	16.4 (6.9–30.9)	25.0 (11.3–43.5)	31.5 (15.5–53.8)	<0.001
Dairy	g/1,000 Kcal	10.3 (0.0–39.5)	38.2 (3.7–87.0)	64.0 (7.5–107.3)	<0.001

The Kruskal–Wallis test was used to test the differences in each characteristic and nutrient intake status according to high, medium, and low MNfA-DI. All variables are presented as median (inter-quartile range). Sugar indicates the total value of glucose, fructose, galactose, sucrose, maltose and lactose. MNfA-DI, Meiji NPS for adults Dietary Index.

Table 2 shows the association between MNfA-DI and each lifestyle-related disease indicator. MNfA-DI was negatively associated with body fat [partial regression coefficient (95% confidence interval) −0.04 (−0.07, −0.01)], diastolic blood pressure [−0.08 (−0.17, −0.002)], fasting plasma glucose [−0.18 (−0.33, −0.01)], and triglyceride levels [−1.36 (−2.16, −0.55)]. Furthermore, Table 3 shows the association between MNfA-DI and various lifestyle-related disease indicators among participants with a BMI between 18.5 and 24.9 kg/m^2^. Except for LDL and HDL cholesterol, significant associations with most variables were confirmed.

## 4 Discussion

This study examined the association between MNfA-DI and each lifestyle-related disease indicator. Higher MNfA-DI was found to be significantly negatively associated with body fat ratio, diastolic blood pressure, fasting blood glucose, and triglycerides. The higher MNfA-DI was also negatively associated with almost lifestyle-related disease indicators when restricted to participants with a BMI between 18.5 and 24.9 kg/m^2^. While the relationship between existing NPS and non-communicable diseases or related disease indicators has been explored worldwide, this study stands as the first to confirm such a relationship specifically within the context of Japan.

A paper published more than 15 years ago suggests that nutritional profiling is not a new system, but many have been developed using a non-systematic and non-logical methodology (52). Different nutrient profiling models are needed for different purposes, but the key requirement is that they are developed using systematic, transparent and logical processes. NPSs with confirmed predictive validity can play an important role in helping companies improve their products to make them healthier (33). For example, leading companies in this field are attempting to reform their product portfolios based on their own developed NPS (33, 36). It also has the potential to make people’s eating habits healthier, as a tool for corporate non-profit initiatives and public health nutrition policies (25). Because the NPS plays a pivotal role in public health nutrition strategies, notably in FoPL. As a measure of strategies targeting these populations, it is not sufficient to assume an association with the disease but to confirm the relationship with the assumed health outcomes, which will increase the validity of the NPS and make it a more effective public health nutrition strategy (38).

The predictive validity of the NPS has been examined mainly in European countries, and the most used measures are the Nutri-Score (Food Standards Agency NPS-DI, FSA NPS-DI) and the HSR. An association with overweight/obesity was reported in a study that used the FSA NPS-DI from the French SUVIMAX cohort (53). A high baseline FSA NPS-DI score (indicative of a poor diet) was associated with increased weight and BMI. Sex differences were also observed between groups. Therefore, FoPL using the FSA NPS may not only contribute to obesity in specific people but may also contribute to effective solutions to the public health population obesity problem through people making conscious food choices. The NutriNet-Santé cohort in France (*n* = 71,403) also supported the association between the FSA NPS-DI and obesity and concluded that it supports the use of the FSA NPS in public policy for chronic disease prevention (54). Additionally, the Spanish PREDIMED-Plus cohort study highlighted the association between FSA NPS-DI and cardiovascular disease risk factors among the older population (55). These findings indicate that a higher baseline FSA NPS-DI correlates with increased levels of several key CVD risk factors, including body weight, fasting plasma glucose, triglycerides, and diastolic blood pressure. However, no association was found between LDL or HDL cholesterol and the FSA NPS-DI, suggesting that while some factors are associated, others are not, highlighting the selectivity of the FSA NPS-DI for health outcomes. The results are quite scientifically interesting, and our findings are similar: the Meiji NPS for adults is not associated with all the key lifestyle-related factors but with fasting blood glucose, triglycerides, and diastolic blood pressure, but not with LDL or HDL cholesterol. Although it is generally accepted that diet intake is associated with LDL and HDL cholesterol by physiological mechanisms, the relationship between diet and LDL or HDL cholesterol is complex and there is no more consistent evidence than for triglycerides or other lifestyle disease indicators (56–59). Consumption of foods high in soluble fiber (e.g., oats, barley) has been negatively associated with LDL-C (although these foods are not commonly eaten in Japan) (60). However, the mechanisms are dependent on eating time and individual socio-economic factors (61, 62). A study using data from 8,344 Japanese subjects (NIPPON DATA90) suggests that SFA and fat per calorie intake are associated with LDL (in men only, the association between fat and HDL is not statistically significant). Thus, following physiological mechanisms, fat or SFA intakes may confirm an association with blood lipids in Japanese (63). However, in the case of the combined scale of carbohydrate, protein, fat, SFA, and the sum of MUFA and PUFA intake, the association between higher scores (indicate the higher intake of fat and SFA) and LDL cholesterol was not significant. This is possible because a high score on this scale indicates a high intake of fat and SFA, but also a low intake of carbohydrate and a high intake of protein and the sum of MUFA and PUFA (64). From a physiological and epidemiological perspective, these studies support the validity of including them as limiting nutrients in the Meiji NPS for adults for the prevention of lifestyle-related diseases. On the other hand, they may explain why epidemiological studies with multiple nutrients do not consistently provide the expected results. As this is a cross-sectional study, longitudinal studies are needed to further clarify the association, especially with LDL and HDL cholesterol.

It is necessary to discuss the development flow of the NPS because the NPS are considered to be strongly limited in their association with health outcomes by pre-defined items. A study that developed a scale to assess the quality of the Japanese diet carefully selected items from several potential scales to assess a healthy diet, considering potential health effects (65). This method is highly dependent on the data used and may need to consider its applicability to different populations. Conversely, the Meiji NPS for adults is data-independent and was developed based on careful consideration of various sources (National Health and Nutrition Survey, Dietary Reference Intakes for Japanese, a large number of Japanese epidemiological studies or meta-analyses, and experts’ comments). While such measures have high applicability in the population, it is necessary to confirm whether the measures are associated with health outcomes. Both are scientific methods, but they differ in their approaches to validation (close to the priori dietary patterns vs. posteriori dietary patterns) (66). This point is also supported by papers discussing the validity of the NPS. While it is important to develop an NPS for people’s health, it is also necessary to confirm its relation to health-related outcomes (23, 38, 39). A scientifically unique NPS, the Food Compass, developed primarily by Tufts University, must be mentioned (67). The Food Compass serves as an NPS for evaluating the healthfulness of a wide variety of foods, beverages, and diets. In a large cohort of US adults, dietary scores derived from the Food Compass were associated with higher BMI, HDL cholesterol, and various disease indicators such as lower systolic and diastolic blood pressure, LDL cholesterol, HbA1c, and fasting plasma glucose levels, suggesting that the Food Compass has a high level of predictive validity. These findings support the relevance of the Food Compass as a tool for guiding public health and private sector strategies to identify and promote healthier diets (68). Furthermore, several other studies have confirmed associations between the Food Compass and inflammatory markers and cardiovascular disease risk and have evaluated its clinical utility (69, 70).

The NutriNet-Santé study utilized weight as an outcome and compared four types of NPS: FSA NPS-DI, Food Standards Australia New Zealand Nutrient Profiling Scoring Criterion, HSR, and the French High Council of Public Health NPS (54). Although the observed differences were small, the feasibility of differentiating between multiple NPS for a single outcome was suggested. Currently, no similar studies have been conducted in Asia; however, it is imperative to evaluate the predictive validity of the Meiji NPS for adults for specific diseases, such as lifestyle-related diseases, in Japan or Asia in future research. Another important feature of the Meiji NPS for adults is modifiable. It has been found that the results of nutritional epidemiological studies derived from observational studies are often updated by time, consumption trends, region, and dietary assessment methods (71–73). In addition, while diet quality is reported to be improving in most parts of the world, Japan has shifted from a diet based on plant foods and fish to a diet based on bread, dairy or animal products, and oil (74, 75). These need to be considered comprehensively and carefully, and important and reliable findings need to be incorporated into algorithms in response to various changes. The Meiji NPS for adults is a scientific NPS formulated based on the concept of an NPS for the adult population of Japan. The application of the Meiji NPS for adults to other regions of the world requires careful consideration, as dietary habits and health issues are likely to be different between east Asia and other regions of the world (76).

### 4.1 Strengths and limitations

The strengths of this study are outlined as follows: Firstly, the accuracy of the variables. The dietary survey employed a three-day food record. Among the data of over 1,000 participants (hundreds of food intake data per person), the dietitian staff checked each type and weight of food. Nutritional values were calculated by referencing standard food tables, resulting in meticulously surveyed data. Additionally, an activity meter was used for step count measurement, which is generally considered to be more accurate than self-reported step counts or step counts obtained from questionnaire surveys. Furthermore, this study’s outcome relied on clinical data derived from actual blood collection and measurements rather than self-reported histories of lifestyle-related diseases. This high accuracy minimizes potential recall and memory biases among participants, thereby enhancing the interpretability of results. Secondly, the study utilized a linear regression model to examine several important confounding factors, such as demographics, socioeconomic status, lifestyle factors, and disease history, thereby enhancing the robustness of the analysis. Thirdly, the MNfA-DI used in this study was calculated using a methodology consistent with previous studies, ensuring methodological continuity and enabling comparisons with earlier research findings. However, this study has also some limitations. Firstly, due to the study design, it was not possible to determine a causal relationship between the MNfA-DI and lifestyle-related disease indicators. As this was the first study in Japan, the primary aim was to identify cross-sectional associations and utilize multiple tables to carefully assess and describe differences in characteristics and nutrient or food intakes as assessed by the Meiji NPS for adults. This descriptive approach is integral to observational studies and is deemed important for evaluating the Meiji NPS for adults. Secondly, the results were limited to NILS-LSA data. As the predictive validity of existing NPS is typically validated using nationally representative data, further discussion is warranted regarding the generalizability of the results of this study. Additionally, the reliability of the study’s findings using nationally representative data must be verified. Nevertheless, various experts carefully collected the NILS-LSA data. The target area has both urban and rural locations and is located almost in the center of Japan, with few peculiar climatic or cultural factors. Consequently, the data collected from people residing in this area are considered to closely resemble the average data for Japan as a whole. Third, although several studies have highlighted the importance of timing of food intake, this study is a hypothesis-driven study design and does not consider chrono-nutrition (77). Finally, this study showing that the Meiji NPS for adults can correctly classify the healthiness of foods and confirm their association with lifestyle-related diseases. However, the actual food choice (purchasing behavior) needs to consider a variety of socio-economic and environmental factors, including sex and age differences (78–80).

## 5 Conclusion

These findings suggest that the Meiji NPS for adults could be associated with a lower risk of lifestyle-related diseases. These findings also support the validity of the Meiji NPS for adults as a measure of food healthiness for the adult population from a public health nutrition perspective. Longitudinal studies are needed to strengthen these findings.

## Data availability statement

The data analyzed in this study is subject to the following licenses/restrictions: The data used in this study are available from the National Institute of Longitudinal Study of Aging (NILS-LSA), but as the data have been used under license for this study, there are restrictions on the availability of these data and they are not publicly available. The data are available from the authors on reasonable request, with permission from the NILS-LSA Data Management Office. Requests to access these datasets should be directed to RO, otsuka@ncgg.go.jp.

## Ethics statement

The studies involving humans were approved by the Ethics Committee on Human Research of the National Center for Geriatrics and Gerontology (approved number: 1757). The studies were conducted in accordance with the local legislation and institutional requirements. The participants provided their written informed consent to participate in this study.

## Author contributions

TY: Conceptualization, Data curation, Formal analysis, Methodology, Software, Validation, Visualization, Writing – original draft, Writing – review & editing. SZ: Data curation, Methodology, Resources, Validation, Writing – review & editing. RW: Methodology, Software, Visualization, Writing – review & editing. TH: Supervision, Validation, Visualization, Writing – review & editing. CT: Data curation, Investigation, Methodology, Project administration, Validation, Writing – review & editing. YN: Data curation, Investigation, Methodology, Project administration, Validation, Writing – review & editing. RO: Conceptualization, Data curation, Funding acquisition, Investigation, Methodology, Project administration, Resources, Software, Supervision, Validation, Writing – review & editing.
